# Repeatability and Reproducibility of Measurements Using a NT-530P Noncontact Tono/Pachymeter and Correlation of Central Corneal Thickness with Intraocular Pressure

**DOI:** 10.1155/2013/370592

**Published:** 2013-10-08

**Authors:** Fusako Fujimura, Kazutaka Kamiya, Kazuko Fujiwara, Nobuyuki Shoji, Kimiya Shimizu

**Affiliations:** ^1^Department of Rehabilitation, Orthoptics and Visual Science Course, School of Allied Health Sciences, Kitasato University, 1-15-1 Kitasato, Minamiku, Sagamihara, Kanagawa 252-0373, Japan; ^2^Department of Ophthalmology, Kitasato University Hospital, 1-15-1 Kitasato, Minamiku, Sagamihara, Kanagawa 252-0373, Japan; ^3^Department of Ophthalmology, School of Medicine, Kitasato University, 1-15-1 Kitasato, Minamiku, Sagamihara, Kanagawa 252-0373, Japan

## Abstract

*Purpose*. To investigate the repeatability and reproducibility of intraocular pressure (IOP) and central corneal thickness (CCT) measurements using a noncontact tono/pachymeter (NT-530P) and to assess the correlation of CCT with IOP. *Methods*. Forty-six eyes of healthy volunteers were measured by two examiners. Three consecutive measurements per eye were performed. Repeatability was assessed using the coefficient of variation, and reproducibility was assessed using Bland-Altman plots. Linear correlations were used to determine agreement between CCT and noncorrected IOP and CCT and corrected IOP, which was calculated using a formula built into the NT-530P. *Results*. The coefficient of variation for IOP was 6.4% and for CCT was 0.4%. The 95% limits of agreement between examiners were −0.17 ± 1.42 mmHg (range: −2.95 to 2.61 mmHg) for IOP, −0.93 ± 4.37 **μ**m (range: −9.50 to 7.64 **μ**m) for CCT. The corrected IOP was significantly higher than the noncorrected IOP (*P* = 0.010.3). The noncorrected IOP significantly correlated with CCT (*r* = −0.4883, *P* = 0.0006). The corrected IOP showed no significant correlation with CCT (*r* = −0.0285, *P* = 0.8509). *Conclusions*. NT-530P offered repeatability and reproducibility in both IOP and CCT measurements. The corrected IOP calculated using the NT-530P was independent of the CCT, suggesting that this IOP may be less influenced by the central corneal thickness.

## 1. Introduction

Intraocular pressure (IOP) plays an indispensable role in the diagnosis and the management of glaucoma. At present, we often use applanation tonometry based on the Imbert-Fick law to measure the IOP error in which central corneal thickness (CCT) is considered to be a major confounding factor. Several studies have found that the IOP values measured using an applanation tonometer were overestimated in eyes with thicker corneas or underestimated in eyes with thinner corneas [1–3]. Accordingly, there is a need for correcting the IOP values using CCT in order to accurately determine IOP by means of the established formulas for correcting IOP values [4, 5]. Since the applanation tonometry is measured by a contact procedure and requires topical anesthesia, there is a possible risk of infection. Moreover, the applanation tonometry can be affected by the hydration state of the cornea. 

A newly developed noncontact tono/pachymeter (NT-530P, NIDEK) has two simultaneous functions, one is as a noncontact applanation tonometer which utilizes an air jet for the measurement of IOP values, which can reduce the possible risk of transmission of infectious diseases, and the other is as a noncontact pachymeter using a rotating Scheimpflug camera for measuring CCT. Both IOP and CCT measurements are noninvasive and do not require topical anesthesia. It can also calculate the corrected IOP using the measurement data of CCT. It is thus expected to offer a simple and useful method for the simultaneous assessment of IOP and CCT in a clinical setting. Since it enables us to perform simultaneous IOP and CCT measurements, and to calculate the corrected IOP by CCT, this instrument is clinically useful for glaucomatous screening. However, to our knowledge, the effect of CCT on IOP measurements has not so far been elucidated using this device. The purpose of the study was two fold, to prospectively investigate the repeatability and reproducibility of IOP and CCT measurements obtained with the NT-530P, and to assess the correlations of CCT with non-corrected or corrected IOP.

## 2. Materials and Methods

### 2.1. Study Population

This prospective study examined forty-six eyes (only the right eye) of 46 healthy volunteers without ophthalmological disease except for refractive error, whose mean age was 31.4 ± 11.2 years (range: 20 to 57 years), whose mean spherical equivalent refractive error was −2.44 ± 2.31 D (range: −6.50 to +1.36 D), and whose mean corneal astigmatism was −1.19 ± 0.65 D (range: −3.00 to −0.25 D). The study was approved by the Institutional Review Board at Kitasato University School of Allied Health Sciences (number 2010-001) and followed the tenets of the Declaration of Helsinki. Written informed consent was obtained from all patients after explanation of the nature and possible consequences of the study.

In all cases, two experienced examiners (A and B) (examiners A and B have 10 and 5 years of experience, resp.,) measured the IOP and the CCT of each eye three times using a NT-530P, and the average value was used for statistical analysis. The volunteer was positioned on the chinrest of the device and asked to look at the target. All measurements by both examiners were performed on the same day at almost the same time. The two examiners took turns randomly in taking measurement. 

### 2.2. Repeatability and Reproducibility

The repeatability of the three measurement values made by examiner (A) was assessed using coefficient of variation (CoV) as a normalized standard deviation (SD), as shown in

(1) $$\begin{array}{c} \;\text{CoV}=\frac{\text{SD}}{\text{mean}}\times 100\left(\%\right).\; \end{array}$$

A smaller CoV means better repeatability. Most authors regard devices with a CoV < 10% as having high repeatability, and a CoV < 5% indicates very high repeatability [6].

 The reproducibility of the measurements made by the two examiners was assessed using the method of Bland and Altman, whereby 95% of the differences or limits of agreement lie between ±1.96 × SD of the mean difference.

### 2.3. Correlation between CCT and IOP

The measurement values obtained by examiner (A) were used to compare the non-corrected IOP with the corrected IOP, the latter of which was calculated using a formula incorporated in the NT-530P. The relationship between CCT and corrected IOP and CCT and non-corrected IOP was evaluated by linear regression analysis.

### 2.4. Comparison of the IOP Values Measured by NT-530P and Goldmann Applanation Tonometry

In order to compare the IOP values measured by NT-530P and the Goldmann applanation tonometry (GAT), we additionally examined twenty eyes (only the right eye) of 20 healthy volunteers without ophthalmological disease except for refractive error, whose mean age was 32.8 ± 9.1 years (range: 22 to 58 years), whose mean spherical equivalent refractive error was −1.70 ± 2.62 D (range: −5.13 to +1.25 D), and whose mean corneal astigmatism was −1.09 ± 0.60 D (range: −2.50 to 0.00 D). In all cases, the IOP of each eye was measured three times using NT-530P and GAT. The between-instrument agreement was assessed by the Bland-Altman method.

### 2.5. Statistical Analysis

The Wilcoxon signed-rank test was used to make comparison between non-corrected IOP and corrected IOP and comparison the IOP values between measured by NT-530P and by GAT. Spearman's rank correlation coefficient test was used to determine the correlations between CCT and non-corrected or corrected IOP. Linear correlations were used to determine agreement between CCT and corrected IOP and that between CCT and non-corrected IOP. The results are expressed as mean ± standard deviation (SD), and a value of *P* < 0.05 was considered statistically significant. 

## 3. Results

### 3.1. Repeatability and Reproducibility

**Figure 1:**
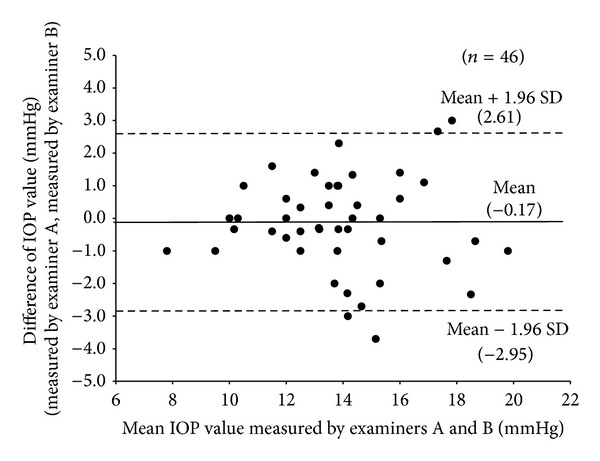
Mean difference (solid line) and 95% limits of agreement (dashed line) for IOP.

**Figure 2:**
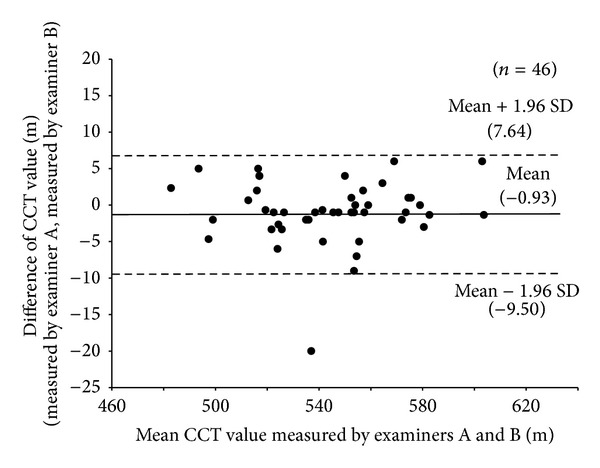
Mean difference (solid line) and 95% limits of agreement (dashed line) for CCT.

The mean and SD of non-corrected IOP acquired by examiner (A) were 13.8 ± 2.6 mmHg, and non-corrected IOP acquired by examiner (B) was 13.9 ± 2.6 mmHg. The mean and SD of CCT acquired by examiner (A) were 543.3 ± 27.9 *μ*m, and CCT acquired by examiner (B) was 544.3 ± 27.8 *μ*m. The coefficient of variation values for non-corrected IOP and CCT was 6.4% and 0.4%, respectively (Table 1). The mean difference (95% limits of agreement) in non-corrected IOP between the two examiners was −0.17 ± 1.42 mmHg (range: −2.95 to 2.61 mmHg) (Figure 1). The mean difference (95% limits of agreement) in CCT between the two examiners was −0.93 ± 4.37 *μ*m (range: −9.50 to 7.64 *μ*m) (Figure 2).

### 3.2. Correlation between CCT and IOP

**Figure 3:**
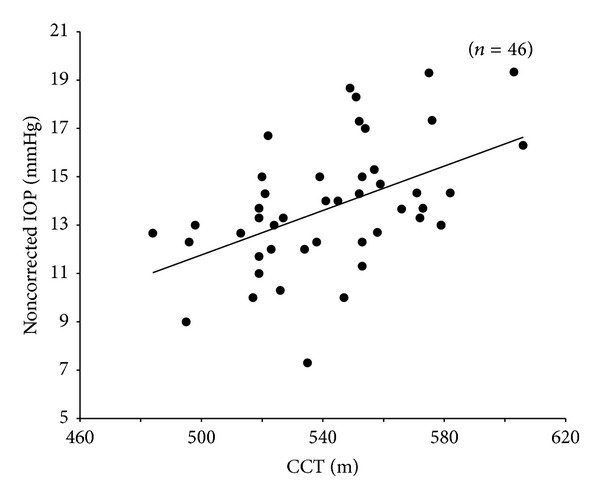
A graph showing a significant linear correlation between the noncorrected IOP and the CCT, *Y* = 0.0459*X* − 11.198; *r* = 0.4883; *P* = 0.0006 (Spearman's rank correlation coefficient).

**Figure 4:**
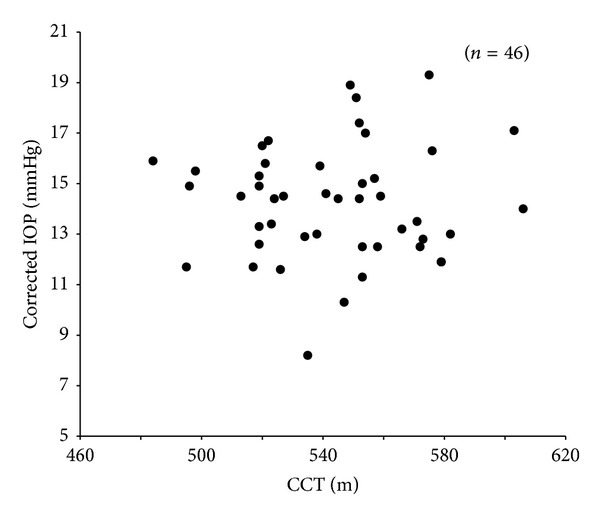
A graph showing no significant correlation between the corrected IOP and the CCT, *Y* = 0.0023*X* + 12.977; *r* = 0.0285; *P* = 0.8509 (Spearman's rank correlation coefficient).

The non-corrected IOP by CCT was 13.8 ± 2.6 mmHg, and the corrected IOP by CCT was 14.2 ± 2.3 mmHg. The corrected IOP was statistically significantly higher than the non-corrected IOP (*P* = 0.0103, Wilcoxon signed-rank test). Moreover, there was a significant positive correlation between the non-corrected IOP and the CCT (*r* = 0.4883, *P* = 0.0006, Spearman's rank correlation coefficient test) (Figure 3). On the other hand, there was no significant correlation between the corrected IOP and the CCT (*r* = −0.0285, *P* = 0.8509) (Figure 4).

### 3.3. Comparison of the IOP Values Measured by NT-530P and Goldmann Applanation Tonometry

**Figure 5:**
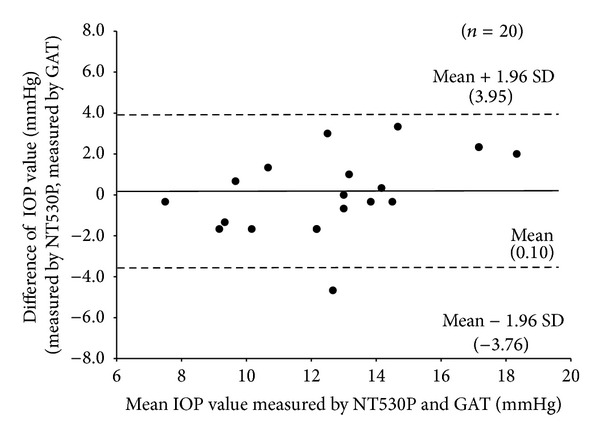
Mean difference (solid line) and 95% limits of agreement (dashed line) for IOP.

The IOP values acquired by NT-530P and GAT were 12.8 ± 3.4 mmHg and 12.7 ± 2.5 mmHg, respectively. There was no significant difference between the IOP values measured by NT-530P and GAT (*P* = 0.2285, Wilcoxon signed-rank test). The mean difference (95% limits of agreement) in IOP between the NT-530P and GAT was 0.10 ± 1.97 mmHg (range: −3.76 to 3.95 mmHg) (Figure 5).

## 4. Discussion

In the present study, the two examiners (A and B) measured the values of the non-corrected IOP and CCT as 13.8 ± 2.6 mmHg (A) and 13.9 ± 2.6 mmHg (B) and 543.3 ± 27.9 *μ*m (A) and 544.3 ± 27.8 *μ*m (B), respectively. The CoV of the IOP measurements was 6.4% and that of the CCT measurements was 0.4%. With regard to the repeatability of IOP measured with the noncontact tonometer, the CoVs of the IOP measurements using NT-2000 and NT-4000 (Nidek) were reported to be 5.6% and 5.2%, respectively [7, 8]. Additionally, Nam et al. investigated the repeatability of CCT measurement with RTVue OCT, Pentacam, and ultrasound pachymetry and reported that the CoVs were 0.31%, 0.64%, and 0.34%, respectively [9]. In this study, the CoVs of IOP and CCT were less than 10%, and the repeatability of IOP and CCT measurements of NT-530P was same or better than that in previous studies. These results indicate that this device has good repeatability to measure IOP and CCT in a clinical setting.

Ogbuehi reported that the 95% limits of agreement for IOP measured in two sessions with the Topcon CT80 noncontact tonometer ranged from −2.45 to 2.65 mmHg, with a mean difference of 0.1 ± 1.3 mmHg [10]. Almubrad reported that the 95% limits of agreement for IOP between two sessions with the GAT and the PT100 noncontact tonometer (Reichert) were −2.0 to 2.1 mmHg and −2.2 to 1.8 mmHg, respectively [11]. The mean difference (95% limits of agreement) in IOP between two examiners in this study was −0.17 ± 1.42 mmHg (range: −2.95 to 2.61 mmHg), which were comparable with that in previous studies. Bourges et al. reported that the 95% limits of agreement in CCT between two examiners with Orbscan 2 and Pentacam were −10.7 to 10.0 *μ*m and −10.0 to 8.6 *μ*m [12]. In this study, the 95% limits of agreement in CCT between two examiners were −9.50 to 7.64 *μ*m, which were the same as those in Bourges' report. Considering the above circumstances, the repeatability and reproducibility of IOP and CCT measurements using this device were excellent. 

Additionally, Ogbuehi et al. reported that there was statically significant difference in IOP readings between RKT7000 (Nidek) and GAT (2.6 ± 2.0 mmHg) and those between CT-80 (Topcon) and GAT (2.8 ± 2.0 mmHg). The 95% limits of agreement between GAT and RKT-7700, and those between GAT and CT-80 ranged from −6.5 to 1.3 mmHg and −6.7 to 1.1 mmHg, respectively [13]. In this study, there was no significant difference between the IOP values measured by NT-530P and GAT. The mean difference (95% limits of agreement) in IOP between the NT-530P and GAT was 0.10 ± 1.97 mmHg (range: −3.76 to 3.95 mmHg). Therefore, we assume that the NT-530P is clinically useful for the simultaneous assessment of CCT and IOP.

In the present study, we found that the corrected IOP by CCT was significantly higher than the non-corrected IOP and that there was a significant correlation between the non-corrected IOP and the CCT, but none between the corrected IOP and the CCT. The formula for correcting IOP in NT-530P was obtained from the results of Burvenich and De Clercq [5] study (2):

(2) Corrected  IOP=measured  IOP+(Standard  CCT−measured  CCT   ×Coefficient  of  adjustment).

The IOP measurements are influenced by the biomechanical properties of the cornea, such as CCT, corneal radius [14, 15], corneal astigmatism [16, 17], and corneal rigidity [18]. The other affective factors (gender, age, and race [3, 19–21], measurement time [22–24], and head location [25, 26]) affect the IOP measurement. Therefore, we cannot acquire an accurate IOP corrected independently by the CCT. In these circumstances, it is necessary to be attentive when interpreting the corrected IOP obtained with NT-530P. 

## 5. Conclusion

Our results indicate that the NT-530P provides excellent repeatability and reproducibility of both IOP and CCT measurements, and that there was a significant correlation between non-corrected IOP and CCT, but none between corrected IOP and CCT. However, we should be aware that there is a possibility of slight IOP overestimation, when the corrected IOP by CCT is used with this device.

## Figures and Tables

**Table 1:** Summary of results obtained from the NT-530P measured by two examiners (*n* = 46 eyes).

	Examiner A	Examiner B	CoV (%)
IOP (mmHg)	13.8 ± 2.6	13.9 ± 2.6	6.4
CCT (*μ*m)	543.3 ± 27.9	544.3 ± 27.8	0.4
